# Phosphate Solubilization Potential of Rhizosphere Fungi Isolated from Plants in Jimma Zone, Southwest Ethiopia

**DOI:** 10.1155/2016/5472601

**Published:** 2016-09-05

**Authors:** Firew Elias, Delelegn Woyessa, Diriba Muleta

**Affiliations:** ^1^Animal Products Veterinary Drugs and Feed Quality Control Assessment Center, P.O. Box 31303, Addis Ababa, Ethiopia; ^2^Department of Biology, Jimma University, P.O. Box 5140, Jimma, Ethiopia; ^3^Environmental Biotechnology Unit, Addis Ababa University, P.O. Box 1176, Addis Ababa, Ethiopia

## Abstract

Phosphorus (P) is one of the major bioelements limiting agricultural production. Phosphate solubilizing fungi play a noteworthy role in increasing the bioavailability of soil phosphates for plants. The present study was aimed at isolating and characterizing phosphate solubilizing fungi from different rhizospheres using both solid and liquid Pikovskaya (PVK) medium. A total of 359 fungal isolates were obtained from 150 rhizosphere soil samples of haricot bean, faba bean, cabbage, tomato, and sugarcane. Among the isolates, 167 (46.52%) solubilized inorganic phosphate. The isolated phosphate solubilizing fungi belonged to genera of* Aspergillus* (55.69%),* Penicillium* spp. (23.35%), and* Fusarium* (9.58%). Solubilization index (SI) ranged from 1.10 to 3.05. Isolates designated as JUHbF95 (*Aspergillus* sp.) and JUFbF59 (*Penicillium* sp.) solubilized maximum amount of P 728.77 *μ*g·mL^−1^ and 514.44 *μ*g mL^−1^, respectively, from TCP (tricalcium phosphate) after 15 days of incubation. The highest (363 *μ*g mL^−1^) soluble-P was released from RP with the inoculation of JUHbF95 in the PVK broth after 10 days of incubation. The present study indicated the presence of diverse plant associated P-solubilizing fungi that may serve as potential biofertilizers.

## 1. Introduction

Phosphorus and nitrogen are the most critical bioelements that limit haricot bean production in Ethiopia [1–3]. Phosphorus (P) is one of the most indispensable macronutrients next to nitrogen for the growth and development of plants [4, 5]. A greater part of soil phosphorus, approximately 95–99%, is present in insoluble form complexed with cations like iron, aluminum, and calcium that cannot be utilized by the plants [6].

The use of natural phosphate-bearing materials such as rock phosphate (RP) as fertilizer for P-deficient soils has received due attention in recent years since substantial deposits of cheaper and low grade RP are locally available in many countries of the world [7]. However, its solubilization rarely occurs in nonacidic soils with a pH greater than 5.5 to 6.0 [8]. Conventionally, RP is chemically processed by reacting with sulphuric acid or phosphoric acid to produce partially acidulated RP. The process incurs high cost and makes the environmental health worse [9–11]. A much cheaper and convenient alternative is reclamation of exhausted soil through use of P-solubilizing microorganisms that have opened the possibility for solubilization of RP in soils. Thus, soil microorganisms play a critical role in natural phosphorus cycle and recently microbial-based approaches have been proposed to improve the agronomic value of RP [12]. This could be another approach for higher cost of manufacturing phosphate fertilizer in industry and also reduces environment pollution posed by a traditional chemical process.

According to Ouahmane et al. [8], utilization of microbial mediated RP has several advantages over conventional chemical fertilizers for agricultural purposes. These advantages are as follows: (1) microbial products are considered safer than many of the chemical fertilizers now in use; (2) neither toxic substances nor microbes themselves will be accumulated in the food chain; and (3) self-replication of microbes circumvents the need for repeated application. Thus, inoculation with phosphate solubilizing microorganisms along with rock phosphate could be another strategy to improve the physicochemical and biological properties of soil and help in improving crop production.

The beneficial plant-microbes interactions in the rhizosphere are determinants of plant health and soil fertility [13]. Among the rhizosphere microbes, the important genera of P-solubilizing bacteria include* Rhizobium*,* Bacillus*, and* Pseudomonas* [14, 15].* Penicillium* and* Aspergillus* spp. are the dominant P-solubilizing filamentous fungi found in rhizosphere [16]. Filamentous fungi are highly important in RP solubilization. They are widely used as producers of organic acid.* Aspergillus niger* and some* Penicillium* species have been tested for solubilization of RP and other biotechnological importance such as biocontrol, biodegradation, and phosphate mobilization [10, 17, 18].

In Ethiopia, effects of plant growth promoting rhizobacteria (PGPR) on growth and yield of tef were evaluated by Woyessa and Assefa [19]. Furthermore, the effect of phosphate solubilizing fungus on growth and yield of tef was studied by Hailemariam [20]. However, information on the diversity of phosphate solubilizing fungi inhabiting various rhizospheres in this region is limited. The present study was therefore designed to isolate and characterize the phosphate solubilizing fungi isolated from various rhizospheric soils.

## 2. Materials and Methods

### 2.1. Description of the Study Area

The study was carried out in Jimma University, Jimma town, located at 353 km to the southwest of Addis Ababa. The microbial analysis was conducted in Postgraduate and Research Laboratory, Department of Biology. Sample collection sites were Jimma town and Manna district of Jimma zone. The total area of Jimma zone is 18415 km^2^ and located between latitudes 7°18′N and 8°56′N and longitudes 35°52′E and 37°37′E. Manna district is one of the woredas in Jimma zone, which is located at 368 km southwest of Addis Ababa. The major soil types of the area are nitosol and combsols and the area receives an average annual rainfall of 1,467 mm per year [21].

### 2.2. Collection of Rhizosphere Soil Sample

A total of one hundred fifty rhizosphere soil samples were collected from 30 plant samples of cabbage (*Brassica integrifolia*), faba bean (*Vicia faba* L.), haricot bean (*Phaseolus vulgaris* L.), sugar cane (*Saccharum officinarum* L.), and tomato (*Lycopersicon esculentum* Mill.). The rhizosphere soil samples were collected from selected kebeles of Jimma town (Becho Bore, Ginjo Guduru, and Awetu Mendera) and Mana district (Sombo Mana, Hunda Toli, Kemise Waraba, Buture, and Gudeta Bula) farmlands of different sites. The kebeles were purposively selected based on the preliminary survey made to identify potential growing areas for the crop. The samples were randomly collected from agricultural fields within 1 to 2 km interval between the same samples. Roots with adhering soils of healthy plants were collected and transferred to sterile plastic bags and transported to the Postgraduate and Research Laboratory, Department of Biology, Jimma University, and stored at 4°C for further analysis.

### 2.3. Isolation of Phosphate Solubilizing Fungi

Collected rhizosphere soil samples were used for the isolation of phosphate solubilizing fungi on Pikovskaya's (PKV) agar medium, containing the following (g/L): 0.5 g (NH_4_)_2_SO_4_, 0.1 g MgSO_4_·7H_2_O, 0.02 g NaCl, 0.02 g KCl, 0.003 g FeSO_4_·7H_2_O, 0.003 g MnSO_4_·H_2_O, 5 g Ca_3_ (PO_4_)_2_, 10.0 g glucose, 0.5 g yeast extract, 15.0 g agar, and 1000 mL distilled water [22]. The medium was autoclaved at 121°C for 15 minutes; about 20 mL of the sterilized molten agar medium was poured into each petri dish and supplemented with 25 *μ*g/mL chloramphenicol to inhibit bacterial growth and allowed to solidify before inoculation.

For each sample, the loosely adherent soils were removed by agitating the roots strongly; the root samples with their adhering soil were cut into pieces (1-2 cm) by sterile scissors and used for isolation. Ten grams of each plant root fragment was aseptically weighed and transferred to 250 mL Erlenmeyer flask containing 90 mL of 0.85% saline solution. The suspension was shaken on 110 rpm for 25 minutes on a rotary shaker and then allowed to settle for 10 min. Aliquots of 1 mL of the supernatant from the sample was transferred to 9 mL of sterile normal saline solution dispensed into test tubes and serially diluted to 10^−1^, 10^−2^, 10^−3^, 10^−4^, 10^−5^, and 10^−6^. From each serially diluted soil suspension, 0.1 mL aliquots were transferred and spread plated on Pikovskaya's agar plates and incubated at 25°C–28°C for 2–7 days. After incubation, fungal colonies showing clear zones around the colonies were further purified by transferring into Pikovskaya's agar medium. The pure cultures were preserved on Potato Dextrose Agar (PDA) slant at 4°C for further investigation.

### 2.4. Identification and Characterization of Phosphate Solubilizing Fungi

PDA was used to accelerate the growth rate and the production of enough conidia as reported by Diba et al. [23]. The characteristics of fresh cultures were compared with mycological identification keys and taxonomic description [24] to identify the isolated fungi to the genus level. Identification was based on colony characteristics and microscopic features, among the colonial characteristics such as surface appearance, texture, and colour of the colonies both from upper and lower side. In addition, conidia, conidiophores, arrangement of spores, and vegetative structures were determined with microscopy. The identified fungi were maintained on Potato Dextrose Agar (PDA) slant at (+4°C) for further investigation. Slide culture was prepared in order to identify spores and mycelia of pure fungal isolates. Accordingly, the morphology of spores and mycelia of fungal isolates was examined and identified by lacto phenol cotton blue staining using microscope and identified after growing them on slide according to Stevens [25].

### 2.5. Screening of Fungi for Phosphate Solubilization

The fungal isolates obtained from rhizospheric soils were evaluated on agar plates and liquid culture media containing sparingly soluble phosphates for their activity in mobilizing phosphate from insoluble sources (tricalcium and rock phosphates).

### 2.6. Determination of Solubilization Index on Solid Medium

All the isolates were screened under* in vitro* condition for their phosphate solubilization activity following the method described by Iman [26] on Pikovskaya's agar medium. A spot inoculation of fungal isolates was made onto the plates in triplicate under aseptic condition and incubated at 25–28°C for 7 days. Uninoculated PKV agar plate served as control. Comparative solubilization index measurement was carried out on day seven of incubation by measuring clear zone and colony diameters in centimeter. Phosphate solubilization index was determined by using the following formula: ratio of the total diameter (colony + halo zone) and the colony diameter [27].

(1) Solubilization  IndexSI=colony  diameter+halo  zone  diametercolony  diameter.

### 2.7. P-Solubilization Efficiency of Selected Isolates in Liquid Media

Based on solubilization index on solid medium, out of all fungal isolates, nine isolates having code numbers JUFbF58, JUToF166, JUCaF37, JUToF167, JUHbF94, JUFbF59, JUFbF60, JUHbF95, and JUCaF38 were selected and further characterized for their efficiency on PKV broth using TCP and RP as inorganic phosphate sources. Accordingly, quantitative estimation of phosphate solubilization activities was carried out in PVK medium amended with tricalcium/rock phosphate for duration of 20 days. Rock phosphate (RP) was kindly obtained from western Wolega zone, Gimbi Woreda, and Bikilal kebele specifically from the area of Tulu Guda Gute. Samples were ground and sieved through 2 mm sieve. The mineral powder was rinsed with distilled water to remove the fine particles.

### 2.8. Preparation of Inocula

Sporulated pure fungal cultures prepared on PVK agar medium were selected for the preparation of spore suspensions from each fungal isolate following the standard procedures of [28]. A total volume of 20 mL sterile water was spread in aliquots on a culture plate from 10-day-old culture and the fungal colony surface was lightly scraped using a sterile glass rod. The cultures were filtered through Whatman number 42 filter paper into a sterile glass bottle. Spore count was done by using a haemocytometer and the suspension of the isolates was adjusted to approximately 10^6^ spores mL^−1^ using sterilized distilled water.

Quantitative estimation of phosphate solubilization was carried out using Erlenmeyer flasks containing 100 mL PVK liquid medium supplemented with 0.5% tricalcium phosphate (TCP) in amounts equivalent to “P” 997 *μ*g/mL [26]. For the comparative study of the ability of RP mobilization, a second medium was prepared replacing 0.5% tricalcium phosphate (TCP) of PVK medium with 0.25% (w/v) rock phosphate in 100 mL^−1^ equivalent to “P” 500 *μ*g/mL with other conditions the same as for TCP solubilization [29]. The initial pH of the medium was adjusted to 7.0 before sterilization.

After sterilization, the medium in each flask was inoculated with the spore suspension of 10% (v/v) of a particular fungal isolate containing 10^6^ spores mL^−1^. Ten milliliters of sterile distilled water inoculated sample was treated as the control. Three replicates were maintained for each test fungus, and mean values were recorded. Incubation was done at 25–28°C in an incubator shaker (Sanco, India) at 150 rpm for 20 days. The amount of Pi released and pH in the broth flasks were estimated at different times (day 5, day 10, day 15, and day 20) in the presence of TCP and RP. An aliquot of 5 mL culture supernatant was aseptically withdrawn periodically from each culture flask at 5-day interval. The samples were spun using the centrifuge (Sigma, Germany) at 5,000 rpm for 25 min to remove any suspended solids and mycelial fragments and supernatant of each culture was analyzed for pH and phosphate concentration. The cultures were filtered through Whatman number 1 filter paper and the filtrates were used for estimation of Pi released. The pH was measured with a pH meter equipped with a glass electrode [28]. The amount of P-solubilized in culture supernatant was estimated using chlorostannous acid reduced molybdophosphoric blue colour method of Jackson [30] and expressed as equivalent phosphate (*μ*g·mL^−1^). 

## 3. Results

### 3.1. Isolation, Characterization, and Screening of PSF Isolates

In this study, a total of 359 fungal isolates were obtained from 150 rhizosphere soil samples from different plants such as cabbage, faba bean, haricot bean, sugar cane, and tomato collected from two districts of Jimma zone, Jimma town and Mana woreda farmlands (Table 1). Out of the isolated fungi, a total of 167 phosphate solubilizing fungal cultures having potential of phosphate solubilization were isolated (Table 1). Of the isolates, the highest numbers of PSF (28.14%) were recovered from tomato and the least (13.17%) was obtained from faba bean (Table 1).

### 3.2. Identification of PSF Isolates

The fungal isolates were characterized and identified as* Aspergillus*,* Penicillium*, and* Fusarium* spp. (Table 2). The isolates displayed diverse morphology and microscopic characteristics as presented in Table 2.

Out of 167 PSF isolated from the rhizosphere soil samples in the present study, 93 (55.69%) species belonged to the genus* Aspergillus* alone followed by* Penicillium* spp. 39 (23.35%) (Table 3). Of all identified,* Fusarium* spp. were the least dominant 16 (9.58%) and the rest 19 (11.38%) fungal colonies were unidentified (Table 3). In addition,* Fusarium* spp. were detected only in the soil samples from rhizosphere of tomato plant, while other identified isolates were common in cabbage, faba bean, haricot bean, sugar cane, and tomato plant root rhizosphere (Table 3).

### 3.3. Qualitative and Quantitative Phosphate Solubilization

The solubilization index (SI) of the isolated phosphate solubilizing fungi ranged from 1.10 to 3.05 at seven days of incubation at 25–28°C (Table 4). Results on (Table 4) revealed that, among the screened 167 PSF isolates, JUCaF38 (*Aspergillus* sp.) was the most efficient phosphate solubilizer on PV plates with SI = 3.05 followed by JUHbF95 (*Aspergillus* sp.) with SI = 2.87 and JUFbF59 (*Penicillium* sp.) with SI = 2.39, whereas the smallest SI of 1.10 was detected from the isolate JUHbPSF61.

**Figure 1 fig1:**
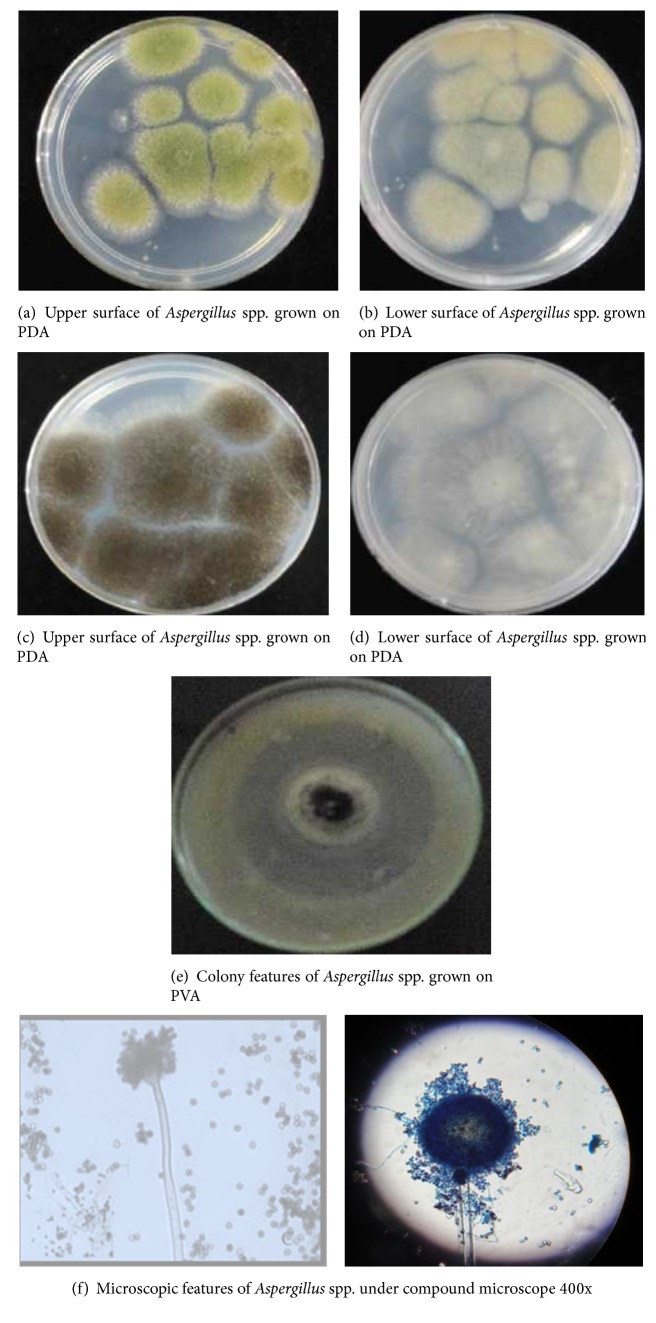
(a), (b), (c), (d), (e), and (f)* Aspergillus* spp. isolated from rhizosphere soil.

**Figure 2 fig2:**
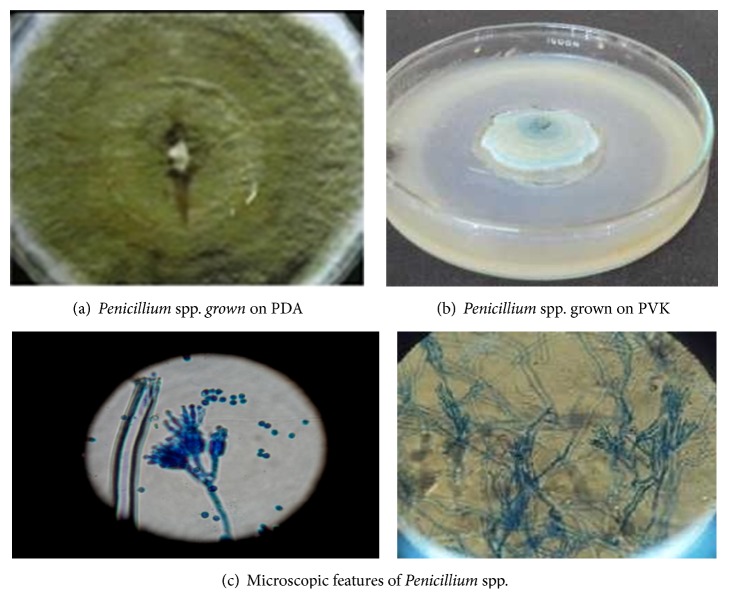
(a), (b), and (c)* Penicillium* spp. isolated from rhizosphere soils.

**Figure 3 fig3:**
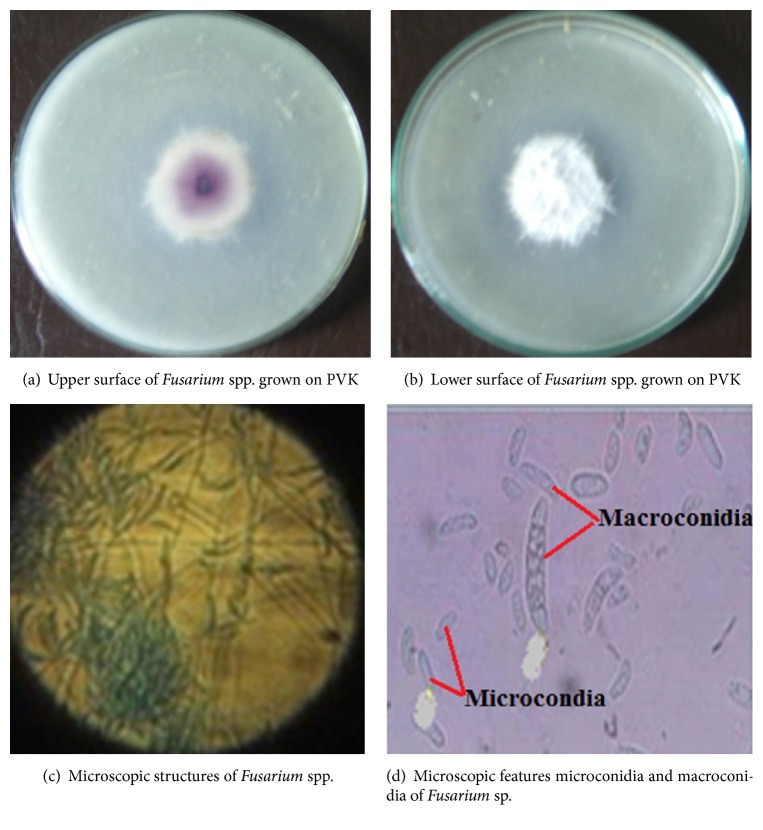
*Fusarium* spp. isolated from rhizosphere soils.

**Figure 4:**
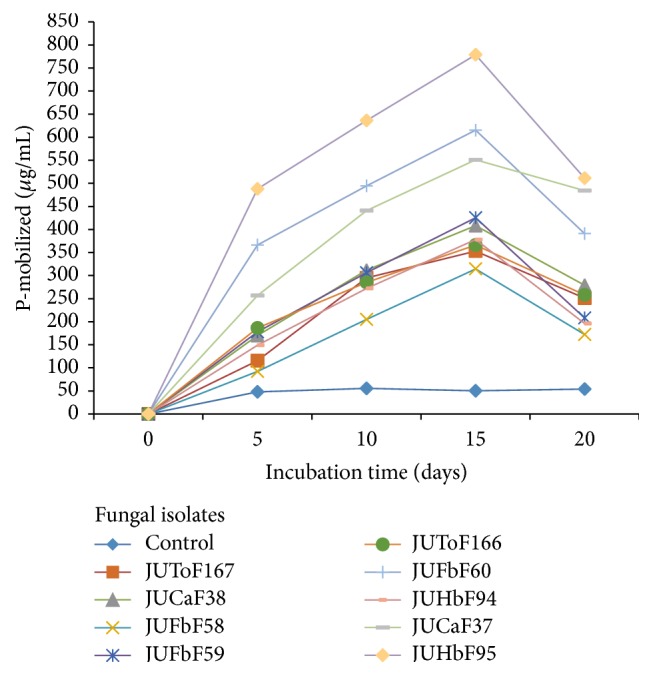
Solubilized P concentrations after 5, 10, 15, and 20 days of incubation in TCP containing PKV broth inoculated with PSF isolates.

The amount of phosphates solubilized by all fungal isolates was showed to be significantly (*p* < 0.05) higher over uninoculated control ([Fig fig1] [Fig fig2] [Fig fig3] Figure 4). The minimum P-solubilized from TCP containing broth was on day 5, afterwards the solubilized P increased up to day 15 of incubation. Accordingly, the mobilized phosphate values in the medium ranged between (92.09–778.77 *μ*g mL^−1^) with variations (*p* < 0.05) among different fungal isolates during 20 days of incubation time. The highest amount of solubilized phosphate (778.77 *μ*g mL^−1^) was recorded from JUHbF95 (*Aspergillus* sp.) inoculated culture filtrates followed by (*Aspergillus* sp.) JUFbF60 (615.40 *μ*g mL^−1^), (*Aspergillus* sp.) JUCaF37 (553.06 *μ*g mL^−1^), and (*Penicillium* sp.) JUFbF59 (472.20 *μ*g mL^−1^). The minimum concentration of soluble-P (225.50 *μ*g mL^−1^) was recorded in the cultures of (*Aspergillus* sp.) JUFbF58 during 15 days of incubation time (Figure 4). In further incubation (at day 20), decline in the mobilized phosphate was recorded in all cases of the test fungal isolates that reached up to 172.36 *μ*g mL^−1^ (minimum value) in case of JUFbF58 and 511.22 *μ*g mL^−1^ (maximum value) in case of the isolate JUHbF95 inoculated culture filtrates (Figure 4).

**Figure 5:**
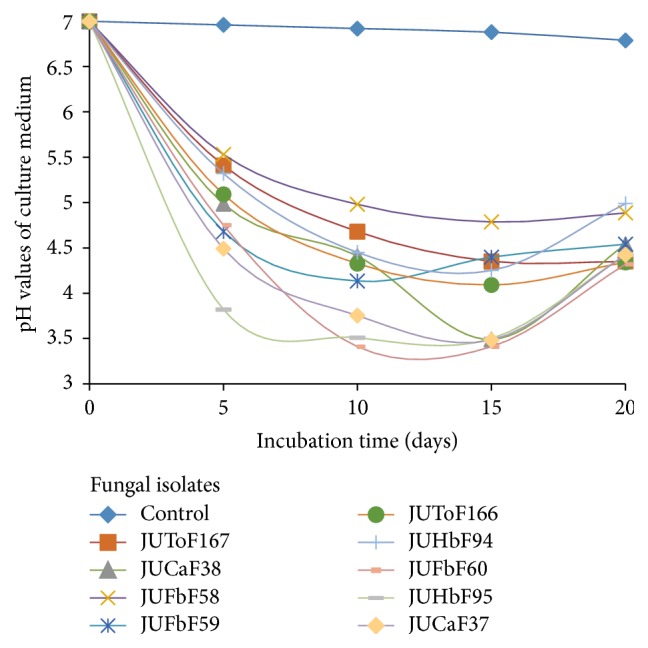
pH values of TCP containing PVK broth inoculated with PSF isolates after 5, 10, 15, and 20 days of incubation.

All the nine isolates showed a decrease in the pH significantly (*p* < 0.05) over control in the liquid culture supernatants during the 20 days of incubation (Figure 5). The pH values decreased to variable levels in the TCP medium during the initial days depending on the culture and later became increased or remained at the same level (Figure 5). The highest drop in pH was recorded in the isolates JUHbF95 and JUFbF60 from initial pH of 7.0 to 3.08 and 3.31, respectively, after 15 days of incubation time.

**Figure 6:**
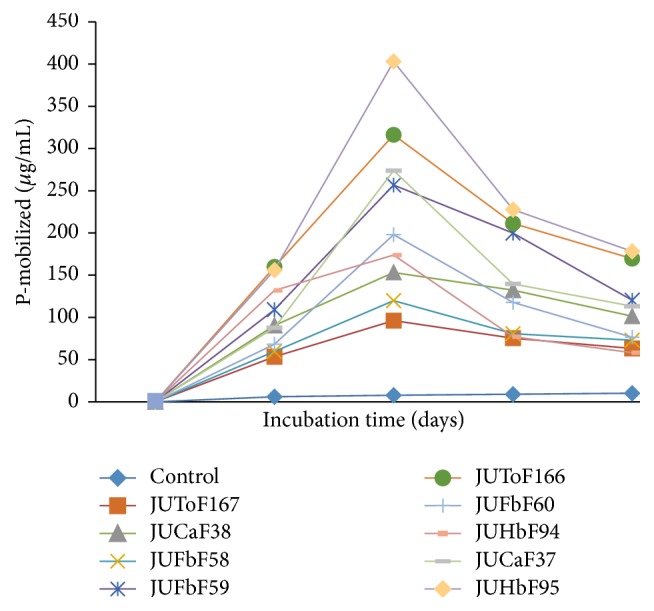
Solubilized P concentrations after 5, 10, 15, and 20 days of incubation in RP containing PKV broth inoculated with PSF isolates.

The pH values at 5, 10, 15, and 20 days of incubation in PVK liquid medium containing RP were summarized in (Figure 6). A highly significant (*p* < 0.05) variation of solubilized P concentrations was recorded among the fungal isolates in 20 days of incubation. The minimum P-solubilized value from RP culture medium during 20 days of RP solubilizing experiments was obtained on day 5 (Figure 6). The results also showed that when the medium was supplemented with rock phosphate, the content of soluble phosphate released by the isolates in culture medium increased up to 10th day in all nine fungal isolates (Figure 6). The highest mobilized phosphate value (363 *μ*g/mL) was recorded from isolate JUHbF95 (*Aspergillus* sp.) whereas the minimum concentration of soluble-P (96.20 *μ*g/mL) was observed in the cultures of JUToF167 (*Aspergillus* sp.) on day 10 of incubation (Figure 6). Mobilized phosphate values began to decline in all inoculated culture filtrates that reached up to 58.17 *μ*g L^−1^ (minimum value) in JUHbF94 inoculated culture and 178.12 *μ*g/mL (maximum value) in case of JUHbF95 on day 20 of incubation time (Figure 6).

**Figure 7:**
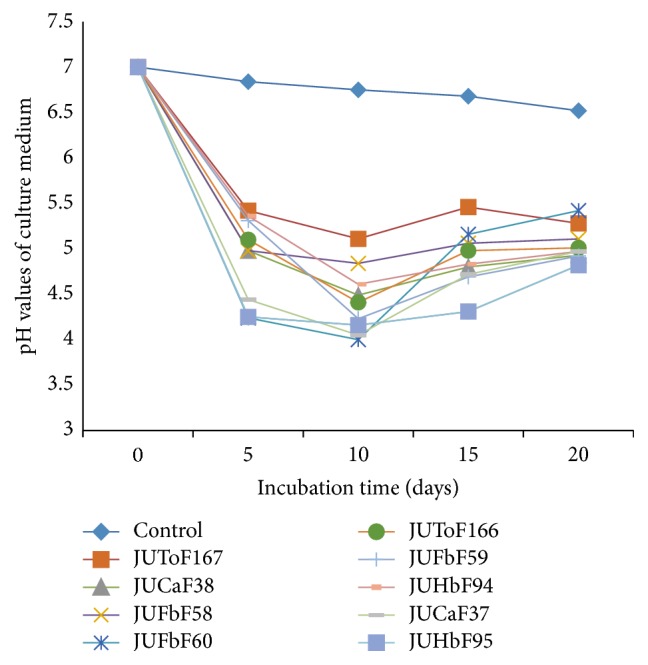
pH values of RP containing PVK broth inoculated with PSF isolates after 5, 10, 15, and 20 days of incubation.

There were significant (*p* < 0.05) drops of pH in the liquid culture medium amended with RP for all the nine test fungal isolates compared to the control during 20 days of incubation (Figure 7). The maximum pH decrease was recorded from the 5th and 10th days of incubations by most fungal isolates but was later increased or nearly constant in all culture filtrates (Figure 7). Among the nine isolates the largest reduction of pH in culture medium was from an initial value of 7.0 to pH values 4.00, 4.05, 4.13, and 4.23 for the isolates JUHbF60, JUCaF37, JUHbF95, and JUFbF59, respectively, after 10 days of incubation (Figure 7). However, no decrement was found in pH medium over the last 15–20 days of incubation in all test culture filtrates.

From nine efficient phosphate solubilizing fungal isolates, two isolates, namely, JUHbF95 (*Aspergillus* spp.) and JUFbF59 (*Penicillium* spp.) resulted in the highest mean phosphate solubilization and mean pH drop from PKV broth containing tricalcium phosphate and rock phosphate, respectively.

## 4. Discussion

The present study revealed that the rhizosphere of different plants collected from Jimma town and Mana district farmlands supports a diverse group of P-solubilizing fungi. The most dominant genera belong to* Aspergillus* and* Penicillium* spp. Similarly, Chuang et al. [10], Oniya et al. [31], and Verma and Ekka [32] also isolated P-solubilizing fungi such as* Aspergillus niger *and* Penicillium *spp. from various rhizospheric soil samples.

In the case of tomato plant rhizosphere soils* Fusarium* spp. along with* Aspergillus *and* Penicillium* spp. were detected. Besides, effective phosphate solubilizing fungi were isolated with higher proportion from tomato plants. In another study, Patil et al. [33] and Yasser et al. [34] isolated P-solubilizing microorganisms from rhizosphere soils. The effectiveness of P-solubilizing fungi in the present study could be probably because of sufficient root exudates since phosphate solubilizing microorganisms are mainly dependent on carbon rich sources from plant root for active production of organic acids that are utilized for solubilizing soil-bound phosphate [35].

In the present study,* Aspergillus* spp. (55.69%) were the most frequently occurring P-solubilizing fungi of the three identified genera. This may be due to the efficiency of* Aspergillus* sp. in root colonization [36]. The current result is in agreement with the earlier findings of several workers [17, 31, 35], who observed predominant occurrence of P-solubilizing fungi belonging to genus* Aspergillus* followed by* Penicillium* spp. in the rhizosphere of different crop plants.

In this study, a total of 19 PSF isolates were reported as unidentified due to overlapping morphological and colonial features. Although morphological and colonial properties are commonly used for identification, molecular, biochemical, and physiological methods are important for the correct identification of fungal species.

The maximum solubilization index was shown by isolates JUCaF38, JUHbF95, and JUFbF59. The variable potential of phosphate solubilization based on SI on agar plate in the present study may be because of the varying type, amount, and diffusion rates of diverse organic acids secreted by fungal isolates as previously reported by Yadav et al. [37]. Iman [26] reported that the solubilization indices (SI) of the test phosphate solubilizing fungal strains (*Penicillium italicum* and* Aspergillus niger*) were 2.42 and 3.15, respectively. Conversely, Mahamuni et al. [38] reported SI for different fungal strains isolated from sugarcane and sugar beet which ranged from 1.13 to 1.59. Alam et al. [39] also reported SI that ranged from 1.53 to 1.80 for the fungal cultures isolated from maize rhizosphere.

Fungal isolates that have shown higher SI on solid agar medium did not show similar trend in liquid broth medium, which is in agreement with the findings of Alam et al. [39] who reported that some isolates with little clear zone on solid agar medium exhibited higher efficiency for dissolving insoluble phosphates in liquid medium. Some fungal isolates showed larger clear zones on agar but low phosphate solubilization in liquid medium. This shows that production of higher SI on solid medium does not necessarily show solubilization efficiency in liquid medium. Thus, the plate technique is insufficient to screen the best P solubilizers and to detect all phosphate solubilizers as commented by Nautiyal [40].

In the present study, periodical estimates of P in broth media revealed the potential of the isolates in releasing P from insoluble phosphate sources. The fungal isolates solubilized the insoluble phosphate sources such as TCP and RP with gradual increase in the middle of incubation period. These results are in agreement with the results reported by Nenwani et al. [36], who have also demonstrated a gradual increase in mobilized P by fungal isolate F1 in liquid cultures. Decrease in phosphate solubilization was observed at the end of incubation time which is in agreement with the findings of Mahamuni et al. [38]. Kim et al. [41] reported that this could be attributed to the availability of soluble phosphate, which had an inhibitory effect on further TCP or RP solubilization, or the depletion of carbon source that limited both the production of organic acids and microbial activity. Another possibility for reduction of mobilized P could be due to the formation of an organo-P compound induced by released organic metabolites, which in turn reduces the amount of available P [42]. Muleta [15] also stated that mobilized P is utilized by fungal cells for their growth and development during this period.

The test fungal isolates solubilized maximum amount of phosphate from TCP on day 15 and from RP on day 10 but gradually decreased afterwards. These results are in conformity with the findings of Pandey et al. [18], who observed maximum solubilization of phosphate occurring at day 15 of incubation for TCP under controlled conditions. Other studies on rock phosphate solubilization indicated that the maximum soluble phosphorus release was on tenth day [11] of incubation by the PSF isolates in liquid culture medium while Vyas et al. [43] reported maximum solubilization of RP from nine to twelve days after incubation. The isolates JUHbF95 and JUFbF60 showed the highest mobilized P values (778.77 *μ*g mL^−1^ and 615.40 *μ*g mL^−1^, resp.) when the medium was supplemented with tricalcium phosphate which could be linked to its inherent self-solubility. These fungal isolates were also able to release considerable amount of P when the medium was supplemented with rock phosphate. This indicates the potential of these fungal isolates in solubilization of insoluble phosphates which gives new avenue for development of fungal biofertilizers after carrying out the necessary qualifying tests. In line with these findings, Chakraborty et al. [44] reported that* Aspergillus niger* (isolate RS/P-14) solubilized maximum amount of tricalcium phosphate (799 *μ*g mL^−1^) and from rock phosphate (385 *μ*g mL^−1^) in PVK broth at 15 days of incubation. Similarly, Pandey et al. [18] recorded mobilized phosphate between 320 *μ*g mL^−1^ (*P. oxalicum*) and 500 *μ*g mL^−1^ (*P. citrinum* and* P. purpurogenum*) from TCP at 15 days of incubation. The tricalcium phosphate was more efficiently solubilized than rock phosphate.

This finding is similar to the earlier studies of Premono et al. [27] and Mahamuni et al. [38] who recorded lesser P solubilization in RP than TCP. These authors remarked that the poor solubilization of rock phosphate may be attributed to the complex mineral composition and particle size in the medium as remarked by Pradhan and Sukla [29] and Mahamuni et al. [38] in addition to the presence of strong apatite bond in the RP, which reduced phosphate solubilization [45].

Acidification by organic acid has been reported to be the main solubilization mechanism of inorganic P by microorganisms [46]. Decrease in pH values was recorded in all fungal isolates differently in the culture media. This might be due to production of diverse organic acids from the available nutrient (glucose) as commented by several investigators [18, 37]. The pH drop in cultures has been repeatedly reported by a number of research findings [18, 37, 40, 47]. The pH values decreased to variable levels depending on the culture type and later became nearly constant or increased with reduction in mobilized phosphate. This observation might be attributed to low glucose concentration in PKV broth which is essential for production of organic acids. In this respect, the present findings are in agreement with Nenwani et al. [36] who reported increase in pH value and decrease in solubilized phosphate at the end of incubation time.

## 5. Conclusions

The results of this study have shown that the rhizosphere soils of cabbage, haricot bean, faba bean, sugar cane, and tomato of different sites of Jimma town and Mana woreda farmlands support a diverse group of naturally occurring potential phosphate solubilizing fungi (PSF). All the selected isolates were capable of mobilizing TCP and RP in PVK broth. The efficiency of phosphate solubilization is significantly higher in Pikovskaya medium containing TCP than in the medium containing RP.

## Figures and Tables

**Table 1:** Total number of PSF isolated from rhizosphere soils of five different plants grown in Jimma town and Mana woreda farmlands.

Source of rhizosphere soil	Number of samples	Number of PSF isolates	Number of other moulds	Proportion of PSF isolates (%)
Tomato	30	47	38	28.14
Cabbage	30	38	42	22.76
Haricot bean	30	35	23	20.96
Sugar cane	30	25	35	14.97
Faba bean	30	22	54	13.17

Total	150	167	192	100

**Table 2:** Colony morphology and microscopic characteristics of the fungal isolates.

Isolates code	Colony morphology	Microscopic observations	Suggested genus
JUCaF (4–15, 27 & 28, 33–38) JUFbF (39–43, 45–55, 60) JUHbF (61–90, 95) JUScF (96, 102–104, 106-107, 117–119) JUToF (136, 143, 148, 152–158, 163–167)	Colonies were initially white and turned yellowish green to light green (Figure 1(a)). Reverse is white to pale green (Figure 1(b)). Colonies grew rapidly on PDA and initially white floccose mycelium spreading rapidly and quickly become black color colonies with production of black spores (Figure 1(c)). Reverse is white to pale yellow (Figure 1(d)).	Conidia were small, black, brownish black, green in colour. Septate hyphae with rough brown and smooth colorless conidiophores with distinctive conidial heads (flask-shaped) (Figure 1(f)).	*Aspergillus *species

JUCaF (1–3, 17–23, 26, 29 & 31) JUFbF (56–59), JUHbF (92 & 94) JUScF (97–100, 105, 108, 112–116 & 120) JUToF (126–129, 135, 142, 144, 159–162)	Colonies are initially white and become dark green or blue green in time with white periphery on both PDA & PVK medium (Figures 2(a) and 2(b)). Reverse was white in color.	Conodia are globuse, greenish and smooth. Septate hyphae, ovate spores and conidial heads composed of continual conidia. Microscopically, conodiophores show branching, and phialides produced in groups from branched metulae, giving brush-like appearance (Figure 2(c)).	*Penicillium* species

JUToF (121–124, 130, 133, 137–140, 143, 145–147, 149, 151)	Colonies grew fast on PDA; the mycelia were floccose, fairly dense, off-white and turned lilac in older portions of the colony. Reverse showed several shades of red to brown on PDA & violet in PVK (Figures 3(a) and 3(b)).	Extensive septate mycelium and conidiophores in the aerial mycelia were mostly short branched (Figure 3(a)). Macroconidia were formed straight, rare and falcate with 2-3 septate per conidium (Figure 3(b)). Microconidia were abundant and occurred singly with oval to obvate shapes (Figures 3(c) and 3(d)) which were fusiform to clavate with, rounded apex, usually with single septa.	*Fusarium* species

JUCaF (16, 24-25, 30 & 32) (JUFbF44), JUHbF (91 & 93) JUScF (101, 109–111) JUToF (125, 131-132, 134, 141 & 150)			Unidentified

**Table 3:** Occurrence of phosphate solubilizing fungi isolated from root rhizosphere soil of different plants collected from Jimma town and Mana woreda farmlands.

Rhizosphere soil samples	*Aspergillus *spp.	*Penicillium *spp.	*Fusarium *spp.	Unidentified
Haricot bean	31	3	0	3
Sugar cane	9	12	0	4
Tomato	15	13	16	6
Cabbage	20	13	0	5
Faba bean	17	3	0	1

Total	93 (55.69%)	39 (23.35%)	16 (9.58%)	19 (11.58%)

**Table 4:** Solubilization index ranges of the fungal isolates on solid Pikovskaya's agar plates.

Isolates code	P-solubilization	
	Number of isolates	SI range
JUCaPSF1–JUCaPSF37	37 (22.12)	1.2–2.40
JUCaPSF38	1 (0.6)	3.05
JUFbPSF39–JUFbPSF60	22 (13.17)	1.24–2.48
JUHbPSF61–JUHbPSF94	34 (20.36)	1.10–2.35
JUHbPSF95	1 (0.6)	2.87
JUScPSF096–JUScPSF120	25 (14.97)	1.16–2.19
JUToPSF122–JUToPSF167	47 (28.14)	1.17–2.32

Total	167	1.10–3.05

Values in parentheses are in percentage; SI = solubilization index.
